# Genetic Structure and Eco-Geographical Differentiation of *Lancea tibetica* in the Qinghai-Tibetan Plateau

**DOI:** 10.3390/genes10020097

**Published:** 2019-01-29

**Authors:** Xiaofeng Chi, Faqi Zhang, Qingbo Gao, Rui Xing, Shilong Chen

**Affiliations:** 1 Key Laboratory of Adaptation and Evolution of Plateau Biota, Northwest Institute of Plateau Biology, Chinese Academy of Sciences, Xining 810001, China; xfchi@nwipb.cas.cn (X.C.); qbgao@nwipb.cas.cn (Q.G.); xingrui@nwipb.cas.cn (R.X.); 2 Qinghai Provincial Key Laboratory of Crop Molecular Breeding, Xining 810001, China

**Keywords:** *Lancea tibetica*, Tanggula Mountains, genetic diversity, population structure, microsatellite markers

## Abstract

The uplift of the Qinghai-Tibetan Plateau (QTP) had a profound impact on the plant speciation rate and genetic diversity. High genetic diversity ensures that species can survive and adapt in the face of geographical and environmental changes. The Tanggula Mountains, located in the central of the QTP, have unique geographical significance. The aim of this study was to investigate the effect of the Tanggula Mountains as a geographical barrier on plant genetic diversity and structure by using *Lancea tibetica*. A total of 456 individuals from 31 populations were analyzed using eight pairs of microsatellite makers. The total number of alleles was 55 and the number per locus ranged from 3 to 11 with an average of 6.875. The polymorphism information content (PIC) values ranged from 0.2693 to 0.7761 with an average of 0.4378 indicating that the eight microsatellite makers were efficient for distinguishing genotypes. Furthermore, the observed heterozygosity (*H_o_*), the expected heterozygosity (*H_e_*), and the Shannon information index (*I*) were 0.5277, 0.4949, and 0.9394, respectively, which indicated a high level of genetic diversity. We detected high genetic differentiation among all sampling sites and restricted gene flow among populations. Bayesian-based cluster analysis (STRUCTURE), principal coordinates analysis (PCoA), and Neighbor-Joining (NJ) cluster analysis based on microsatellite markers grouped the populations into two clusters: the southern branch and the northern branch. The analysis also detected genetic barriers and restricted gene flow between the two groups separated by the Tanggula Mountains. This study indicates that the geographical isolation of the Tanggula Mountains restricted the genetic connection and the distinct niches on the two sides of the mountains increased the intraspecific divergence of the plants.

## 1. Introduction

High-mountain zones experience long-term uplift and climatic fluctuations, which result in a variety of evolutionary processes leading to high levels of taxon richness and rarity [1,2]. This is the case in the Qinghai-Tibetan Plateau (QTP), which has the highest biodiversity on the planet [3] with more than 12,000 species of vascular plants distributed in this region, belonging to 1500 genera, accounting for 34.3% of the total species and 50% of the total genus of vascular plants in China [4]. The uplift of the QTP began in early Eocene due to the initial collision between the Asian and Indian plates, and the rapid uplifting of the QTP started in the late Pliocene and/or the early Quaternary [5,6]. The uplift of the QTP has extensively changed the climate and natural geographical environments of China and Asia [7] and has also had a profound impact on plant genetic diversity in this region [8]. The uplift of the QTP formed geographical barriers to restrict species gene flow and promoted plant exotic differentiation [9,10,11]. Meanwhile, the uplift coupled with climatic changes led to niche diversity, which caused rapid radiation and evolution of plants in this region [12,13]. Beside the uplift-driven diversification, four major glacial alternations occurred during the uplift and no large unified ice-sheets were formed, which had a significant impact on the genetic diversity pattern of the species [14,15,16].

Complex and diverse genetic diversity patterns are evident in the QTP due to the long and complicated plateau uplift history and periodic glacial alternations, and the intrinsic characteristics of different species (e.g., generation time, habitat adaptability and distribution range) [17,18]. In the eastern section of the QTP and adjacent areas, orogeny in the Hengduan Mountains led to genetic differentiation and speciation and this was exacerbated by periodic glaciations. Indeed, growing evidence shows that this region was a main refugium for a large number of alpine species [17]. In the northwest region of the QTP and adjacent areas (Altai and Tianshan ranges), orogeny and drought in central Asia promoted the diversity and speciation of species [19,20]. In the central platform of the QTP region, few studies have been conducted, but intraspecific divergences due to geographical barriers can still be found in *Rhodiola alsia* [21], and *Stuckenia filiformis* [22]. In this region, the terrain is relatively flat, but the high-altitudinal mountains covered with snow and ice all year round act as a geographic barrier for gene flow between populations on a regional scale [17]. For instance, the Tanggula Mountains lie east to west in the central QTP with a mean altitude over 5000 m, and they have unique physical and geographical significance. The north side and the south side of the Tanggula Mountains have obvious differences in geomorphology, climate, and vegetation [23], which induce distinct ecological niches. Studies have reported that if related species live in significantly different niches, ecological divergence is likely be important in facilitating speciation, even in the presence of gene exchange [24,25,26]. So, the Tanggula Mountains are an ideal model for testing the effects of geographic barriers on population differentiation, and until now, its effects on the plant species differentiation have not been fully studied. In this study we seek to better understand whether the Tanggula Mountains act as a gene flow barrier leading to the genetic diversity of QTP alpine species.

*Lancea tibetica* is a perennial herb belonging to the Mazaceae family [27] and is mainly distributed in grasslands, sparse forests, and ravines of Tibet, Qinghai, Gansu, Sichuan, and Yunnan with an altitude of 2000–4500 m. It is also found in India, Bhutan and Nepal [28]. As a traditional Tibetan medicine, *L. tibetica* is widely used in the treatment of pneumonia, asthma, cough, carbuncles, colic, and even heart disease [29], because it contains abundant lignans, phenylpropanoids, terpenoids, flavonoids, and steroids [30,31,32,33,34]. However, the wide usage and expanded collection of this species has induced a drastic reduction of the wild resources [35].

*Lancea tibetica* was selected as the ideal study species, not only because this species is a widely distributed species on the QTP, especially its distribution range across the Tanggula Mountains, but also because the seeds are small and wingless and their dispersal capacity is limited, a trait that enhances the degree of genetic differentiation by restricting gene flow. In order to test the hypotheses mentioned above, eight microsatellite markers were used to determine the genetic diversity and population structure patterns of 31 wild populations of *L. tibetica* and to elucidate if these genetic differentiations correlated with environment gradients. Such information contributes to the understanding of the distribution shift of alpine plants caused by geographical changes in the QTP.

## 2. Materials and Methods

### 2.1. Plant Materials

In total, 456 *L. tibetica* individuals from 31 wild populations were collected. Populations were separated from each other by at least 50 km, and the sampling was performed in Qinghai, Gansu, Sichuan, and Tibet (Table S1). Individuals from the same population were spaced at least 50 m apart. Fresh leaves were collected and dried in silica gel. We deposited the voucher specimens in the Herbarium of the Northwest Institute of Plateau Biology (HNWP), Chinese Academy of Sciences, Xining, Qinghai Province, China.

### 2.2. DNA Extraction and SSR Amplification

Total DNA was extracted from dried leaves of 456 samples using a modified cetyltrimethylammonium bromide (CTAB) method [36]. The quality of the DNA was checked by electrophoresis using 1.0% agarose gels and the quantity of the DNA was measured using a NanoDrop spectrophotometer (Thermo Fisher Scientific, Carlsbad, CA, USA).

Eight pairs of microsatellite markers developed by Tian et al. [35] were used in the present study (Table 1). PCR reaction was performed in 20 μL reaction mixture containing 20 ng of template DNA, 2 μL of 10× PCR buffer (15 mM MgCl_2_), 0.5 μL of each primer (5 pM), 0.2 μL of Taq DNA polymerase (TaKaRa Biotechnology Co., Dalian, China), and 0.5 μL of dNTP mix (10 mM), supplemented with ddH_2_O. The PCR reaction conditions included an initial denaturation (94 °C for 5 min), followed by quantification for 40 cycles (94 °C for 30 s, an appropriate primer specific annealing temperature for 35 s, 72 °C for 60 s), and a final extension (72 °C for 10 min). The amplified products were size separated by capillary electrophoresis on an Applied Biosystems Genetic Analyzer (ABI 3730) from Sangon Biotech Co., Ltd. (Shanghai, China) to obtain the size of microsatellite fragments.

### 2.3. Data Analysis

POPGENE 1.32 (https://sites.ualberta.ca/~fyeh/popgene.html) was used to determine the genetic diversity parameters such as observed number of alleles (*N_a_*), effective number of alleles per locus (*N_e_*), observed heterozygosity (*H_o_*), Nei’s expected heterozygosity (*H_e_*), Shannon information index (*I*), fixation index (*F_st_*), and the inbreeding coefficient (*F_is_*) of eight microsatellite loci. The polymorphism information content (PIC) of the microsatellite marker was calculated using PowerMarker 3.25 (https://brcwebportal.cos.ncsu.edu/powermarker/). Additionally, Nei’s coefficient of genetic differentiation (*G_ST_*) and Shannon’s coefficient of genetic differentiation (*G_ST_’*) was calculated using a Microsatellite Analyzer (MSA) 4.05 (http://i122server.vu-wien.ac.at/MSA). In the current study, we hypothesized that the population patterns conformed with the island model, and the gene flow (*N_m_*) can be calculated based on the following formula:

Nm = 0.25 (1−Fst)/Fst

To further analyze the genetic relationship among 31 populations, Nei’s genetic distance matrix was calculated using POPULATIONS 1.2.28 (http://bioinformatics.org/populations/). A Neighbor-Joining (NJ) tree was constructed by MEGA 7.0 (https://www.megasoftware.net/) based on Nei’s genetic distance matrix. Meanwhile, the principal coordinate analysis (PCoA) of 31 populations was carried out using the Multi-Variate Statistical Package (MVSP) 3.13 (Kovach Computing Services, Pentraeth, United Kingdom).

We evaluated the genetic structure using Bayesian model-based clustering in STRUCTURE 2.3.4 (https://web.stanford.edu/group/pritchardlab/structure.html). The population structure was detected under both the admixture model and no admixture model at the same time, with a burn-in period set at 100,000 and Markov Chain Monte Carlo (MCMC) repetitions after burn-in set at 100,000. The optimal *K* value was determined based on the change in slope of the plot of Ln Pr(*X*|*K*) versus Δ*K*, which is generally the corresponding *K* value at the inflection point of the curve.

Analysis of molecular variance (AMOVA) was performed using ARLEQUIN 3.5 (http://cmpg.unibe.ch/software/arlequin35/) after populations were grouped by STRUCTURE model-based and geographic regions of genetic diversity. Molecular variance was analyzed at two hierarchical divisions, within and among populations. Pairwise comparison of *F_st_* values between the populations was conducted in GENEPOP 4.0 (http://www.genepop.curtin.edu.au/). We investigated the historical barriers to gene flow among collection sites using Monmonier’s maximum difference algorithm in the software BARRIER 2.2 (http://ecoanthropologie.mnhn.fr/software/barrier.html).

To investigate the correlation between genetic distance and geographical distance, a geographic distance matrix between 31 populations was calculated in Microsoft Excel. The obtained geographic distance was tested against genetic distance by the Mantel test in ARLEQUIN 3.5 (http://cmpg.unibe.ch/software/arlequin35/).

## 3. Results

### 3.1. SSR Markers and Genetic Diversity

We employed eight microsatellite markers to genotype 456 individuals of *L. tibetica* from 31 populations. Overall, 55 alleles were amplified, ranging from 3 (LT7) to 11 (LT10, LT25) alleles per locus with an average of 6.875. We identified 34 rare alleles (RA) with a frequency less than 0.5% in seven microsatellites (except LT7), ranging from one to nine per locus with an average of 4.25 and accounting for 61.8% of all the alleles. The Shannon information index (*I*) varied from 0.6333 (LT15) to 1.8425 (LT25) (mean = 0.9394). The observed heterozygosity (*H_o_*) ranged from 0.1752 (LT4) to 0.9978 (LT16) with an average of 0.5277. The Nei’s expected heterozygosity (*H_e_*) ranged from 0.2760 (LT4) to 0.8008 (LT25) with an average of 0.4949. The inbreeding coefficient at the population level (*F_is_*) ranged from −0.9328 (LT16) to 0.0771 (LT4) with an average of −0.2802, and *F_is_* >0 was observed only in LT4 and LT25. The proportion of differentiation among populations (*F_st_*) ranged from 0.0034 (LT16) to 0.4180 (LT25) with an average of 0.2215. In addition, the inbreeding coefficient at the total sample level (*F_it_*) ranged from −0.9262 (LT16) to 0.3850 (LT28) with an average of −0.0581. The polymorphic information content (PIC) for each marker was computed, and it varied from 0.0971 (LT18) to 0.7761 (LT25) with an average of 0.3727. The Nei’s genetic differentiation coefficient (*G_ST_*) ranged from 0.0075 (LT16) to 0.3908 (LT28) with an average of 0.2075. Shannon’s coefficient of genetic differentiation (*G_ST_’*) was consistent with *G_ST_*. The highest value was observed in LT28 (0.5328) and the lowest in LT16 (0.0157), with an average of 0.3406. Based on *F_st_* calculation, gene flow (*N_m_*) ranged from 0.3481 (LT28) to 72.932 (LT16) with an average of 0.8832.

Genetic diversity of 31 populations was evaluated based on the amplified polymorphic alleles (Table S2). The observed number of alleles per locus (*N_a_*) ranged from 1.75 in DL to 3.875 in JD, with an average of 2.65. Meanwhile, the effective number of alleles per locus (*N_e_*) varied from 1.4419 in GH to 2.4170 in LZ, with an average of 1.8798. The Shannon information index (*I*) varied from 0.3763 (GH) to 0.8751 (LZ) with a mean of 0.6536. The percentage of polymorphic loci (PPL) ranged from 62.5% to 100% with an average of 89.5%. The observed heterozygosity (*H_o_*) and the expected heterozygosity (*H_e_*) ranged from 0.4195 (GH) to 0.6562 (LZ, TR) and 0.2453 (GH) to 0.5091 (MLS), with an average of 0.5343 and 0.3954, respectively.

### 3.2. Population Structure

**Figure 1:**
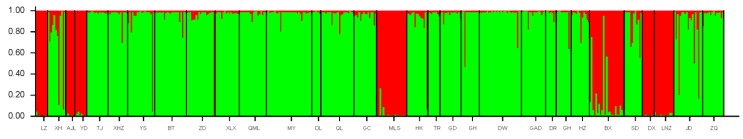
Results of grouping in *Lancea tibetica* by STRUCTURE.

Based on the microsatellite data, the STRUCTURE group calculation was performed, and the change in slope of Ln Pr(*X*|*K*) value and Δ*K* value were plotted (Figure S1). Both Ln Pr(*X*|*K*) and Δ*K* values had obvious inflection points with change in *K* value, and the best *K* value was 2. This supported the division of *L. tibetica* into two groups (*K* = 2). According to the recommended *K* values, STRUCTURE groupings are shown in Figure 1. The first group consisted of 86 individuals from 7 populations: LZ, AJL, YD, MLS, BX, DX, and LNZ, and the second group consisted of 370 individuals from 24 populations: XH, TJ, XHZ, YS, BT, ZD, XLX, QML, MY, DL, QL, GC, HK, TR, GD, GH, DW, GAD, DR, HN, HZ, SD, JD, and ZQ.

**Figure 2:**
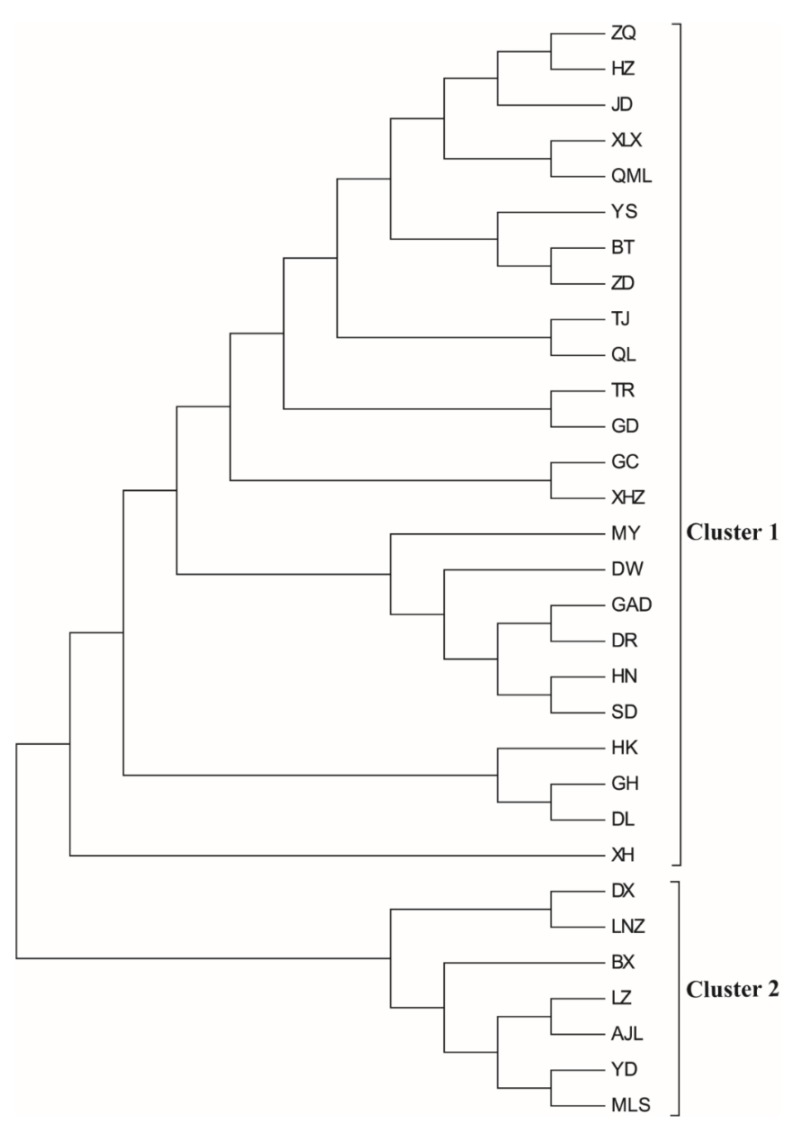
Neighbor-joining (NJ) tree of *L. tibetica* populations based on genetic distance.

For studying the genetic structure of *L. tibetica*, NJ clustering analysis was conducted to estimate the genetic relationships among the 31 populations using Nei’s genetic distance matrix (Figure 2). The results showed that these populations were classified into two main clusters, which were consistent with the results of STRUCTURE grouping (*K* = 2).

**Figure 3:**
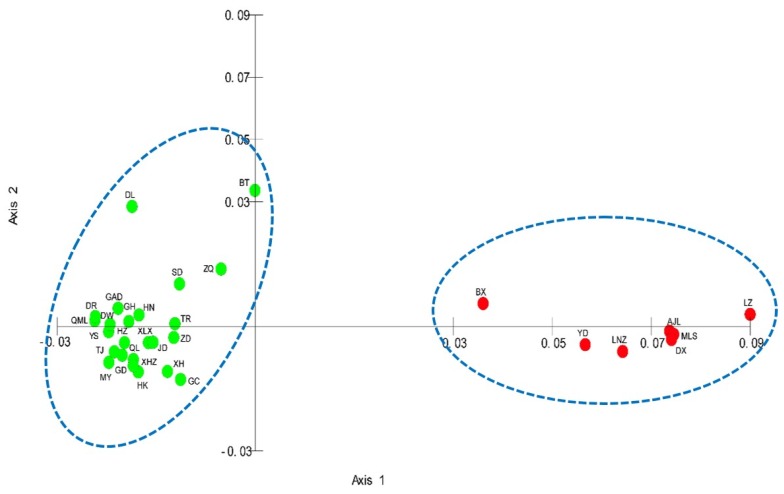
Results of principal coordinates analysis.

To investigate the population structure, principal coordinate analysis (PCoA) was carried out and a scatter plot was generated based on the entire microsatellite dataset of 31 populations. The first and second principal coordinates explained 84.188% and 8.216% of the molecular variance. The PCoA results indicated the division of 31 populations into two distinct groups (Figure 3). LZ, AJL, YD, MLS, BX, DX, and LNZ populations gathered together on the left side of the plot, while the remaining 24 populations gathered together on the right side of the plot. This result was consistent with the STRUCTURE analysis (*K* = 2) and NJ tree.

### 3.3. Genetic Differentiation

In order to estimate the partitioning of genetic variation, an AMOVA based on microsatellite data was conducted (Table 2). The analysis revealed that the main genetic variation in *L. tibetica* was within the populations (81.6839%) rather than between populations (18.3161%). AMOVA was also performed on the two groups obtained by the population structure analysis. The results revealed that 20.9305% of the genetic variance occurred between the two groups, while 9.0187% of the genetic variance occurred within groups among populations. Furthermore, 70.0508% of the genetic variance occurred within populations.

**Figure 4:**
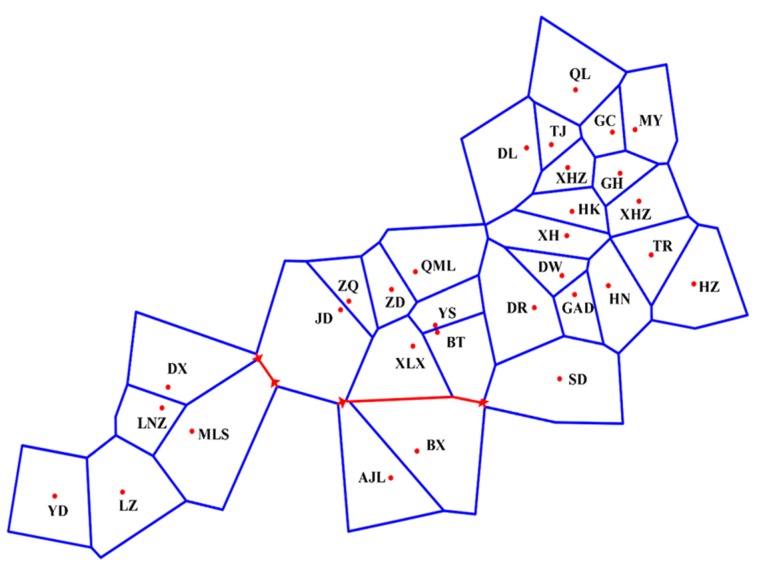
Genetic break among *L. tibetica* populations detected by BARRIER (Numbers of barriers = 2).

Genetic barriers among 31 populations were predicted using Monmonier’s maximum difference algorithm on BARRIER 2.2. When the number of barriers was one, BX and AJL were separated from all other populations. When the number of barriers was two, YD, LZ, MLS, LNZ, and DX were further separated (Figure 4). This was consistent with the results of STRUCTURE grouping, NJ tree, and PCoA. Furthermore, the first and the second barrier separated the populations in Tibet from populations in Qinghai, Gansu, and Sichuan.

The pairwise *F_st_* and *N_m_* for 31 populations were also calculated (Table S3). The results revealed the highest genetic differentiation (*F_st_* = 0.5222) and lowest gene flow (*N_m_* = 0.2287) between DL and AJL. Meanwhile, the lowest genetic differentiation (*F_st_* = 0.0011) and highest gene flow (*N_m_* = 227.0227) were between DX and LNZ.

### 3.4. Genetic Diversity Associated with Geography

**Figure 5:**
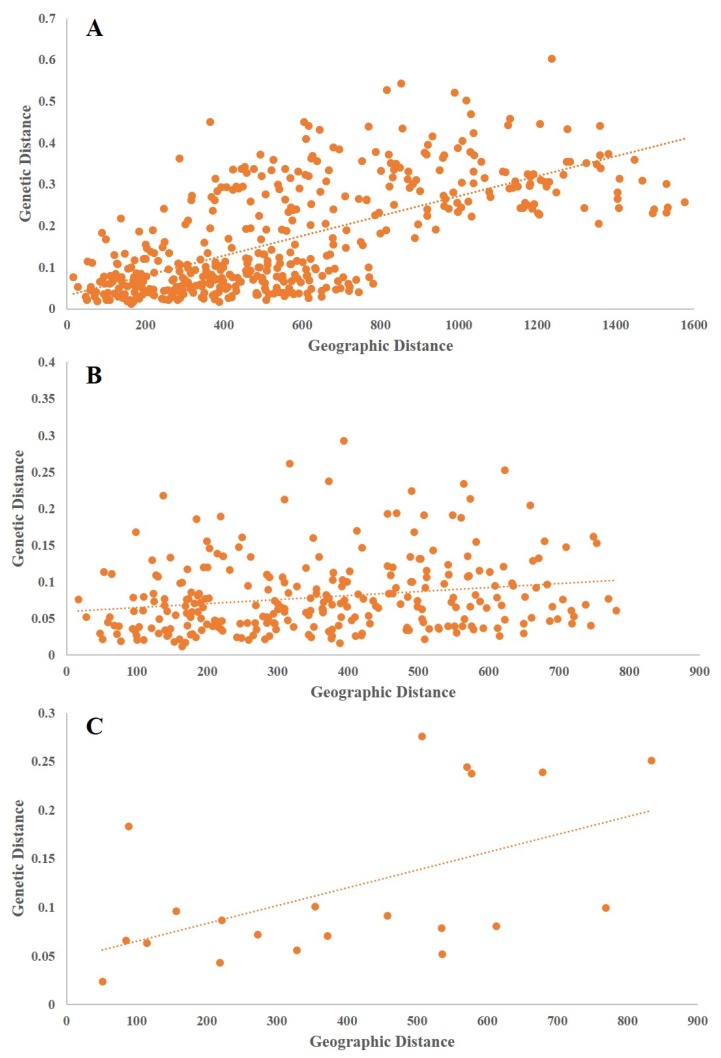
Results of Mantel test between genetic matric and distance matric in *L. tibetica*. (**A**) Mantel test on all populations, *R^2^* = 0.6702, *p* = 0; (**B**) Mantel test on the northern branch populations, *R^2^* = 0.2018, *p* = 0012; (**C**) Mantel test on the southern branch populations, *R^2^* = 0.5324, *p* = 0.004.

**Figure 6:**
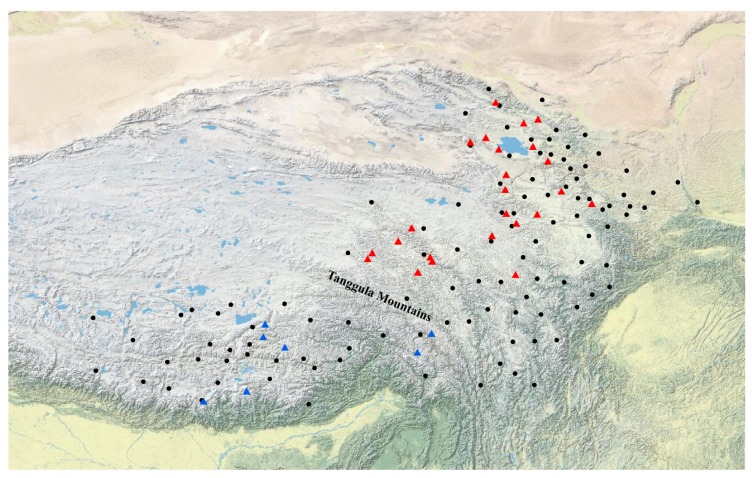
Geographic distribution map of *L. tibetica* (black dots represent herbarium records in the Chinese Virtual Herbarium, the red triangles represent the northern branch, the blue triangles represent the southern branch).

We performed a Mantel test on all the populations that revealed a significant correlation between genetic distance and geographical distance (*R^2^* = 0.6702, *p* = 0; Figure 5A). When the Mantel test was performed on the northern branch, a weak correlation between genetic distance and geographical distance (*R^2^* = 0.5324, *p* = 0.004; Figure 5B) was detected, while a high correlation was found in the southern branch (*R^2^* = 0.2018, *p* = 0.012; Figure 5C). Sampling information of 31 *L. tibetica* populations projected onto a map using ArcGIS 10.2 and the results of genetic grouping are shown in Figure 6, which shows the division of 31 populations into two distinct branches. The populations from Tibet formed a southern branch and the populations from Qinghai, Gansu, and Sichuan formed a northern branch. The boundary between the northern and the southern branches was roughly located in the line of the Tanggula Mountains.

## 4. Discussion

Genetic diversity is the total number of genetic characteristics in the genetic makeup of a species that serve as a means for populations to adapt to changing environments. Rich genetic diversity can help maintain species diversity and stability [37]. It can also slow down the extinction process caused by adaptation and evolution [38]. Microsatellite markers are efficient in examining genetic diversity and exploring genetic relationships in plants, and have been widely used to investigate the genetic diversity of Qinghai-Tibetan Plateau species [39,40,41,42,43,44,45].

### 4.1. Genetic Variation

In the current study, a rich genetic variation was detected in *L. tibetica* (*N_a_* = 6.875, *H_o_* = 0.5277, *H_e_* = 0.4949, *I* = 0.9394). The high level of genetic diversity within the populations of *L. tibetica* is similar to other species that are distributed in the QTP (e.g., *Elymus nutans, H_e_* = 0.719 [44]; *Armillaria luteovirens, H_e_* = 0.521 [45]; *Sibiraea laevigata, H_e_* = 0.834 [46]; *Sibiraea angustata, H_e_* = 0.832 [46]; *Stipa purpurea, H_e_* = 0.585 [47]; *Carex moorcroftii, H_e_* = 0.579 [48]). The high genetic diversity of *L. tibetica* may be due to its biological characteristics such as perenniality, pollination and seeds characteristics. In addition, a relatively low gene flow among populations (*N_m_* = 0.8823) might also result in a high level of genetic diversity. *F_is_* and *F_it_* values indicated heterozygote excess in *L. tibetica* populations, suggesting a heterozygous advantage. This may be attributed to the fact that heterozygous genotypes grow faster and have lower mortality than homozygous genotypes, resulting in higher population heterozygosity [49]. The high genetic diversity of alpine species in the QTP gives rise to some degree of adaptation to their respective environmental conditions.

### 4.2. Genetic Divergence and the Effects of the Tanggula Mountains

Mantel tests showed that isolation by distance had an important impact on the genetic divergence of *L. tibetica*. Meanwhile, Bayesian clustering (STRUCTURE), NJ cluster analysis, PCoA and genetic barrier prediction analysis demonstrated that the Tanggula Mountains played a significant role in intraspecific divergence. All the populations can be divided into two main groups: the northern branch and the southern branch, roughly bounded by the line of the Tanggula Mountains. A similar pattern was also detected in previous studies of *Rhodiola alsia* [21] and *Stuckenia filiformis* [22] by using nuclear ITS sequence and chloroplast sequences. The pairwise *F_st_* and *N_m_* demonstrated that gene flow between populations on each side of the Tanggula Mountains is greater than across the Tanggula Mountains. Take MLS and JD for example, the two populations were located on different sides of the Tanggula Mountains, and the geographic distance was not very great, but a high genetic differentiation (*G_ST_* = 0.2710) and low gene flow (*N_m_* = 0.6722) was detected. The gene flow between them is less than that between populations on the same side of the mountains. The genetic divergence pattern strongly suggests that the Tanggula Mountains might be a geographic barrier that restricts the gene flow between the populations on the different sides of the Tanggula Mountains.

The genetic divergence of *L. tibetica* in the Tanggula Mountains can mainly be attributed to the snow-cover on the ridges of the mountains. High altitude snow-covered ridges can directly obstruct the genetic connection and reduce the distance of transmission. The differences in the ecological niches on the two sides (north and south) also play an important role. In terms of geomorphology, the northern side is relatively flat with an intact plateau environment and the headward erosion of modern rivers is not obvious, while the topography of the southern side is relatively fragmented forming mountains and gorges, and the rivers are severely cut [50]. Meanwhile, the climate in the northern region, influenced by the Asian monsoon is cold, dry, and has less precipitation, while in the southern region the climate is mainly influenced by the Indian Ocean monsoon, and is warmer and highly humid with relatively more precipitation [51,52,53]. In terms of vegetation, alpine shrub meadow, cold alpine steppe, and cold alpine desert steppe are mainly found on the northern side, while mountain forest, mountain shrub steppe, and alpine steppe are mainly distributed on the southern side [54]. Such distinct ecological niches reinforce the divergence of the two lineages following their initial spatial isolation.

## 5. Conclusions

This is the first study presenting the genetic diversity of *L. tibetica* based on microsatellite markers. The study revealed the significant role of the Tanggula Mountains in determining the genetic diversity and population structure of *L. tibetica*. Geographical barriers restricted gene flow among different populations and resulted in intraspecific genetic divergence. Genetic structure and eco-geographical differentiation can maintain adaptation to continuous geographical and environmental change.

## Figures and Tables

**Table 1:** Genetic characteristics of eight microsatellite loci in *Lancea tibetica* populations. Observed number of alleles (*N_a_*), rare alleles (*RA*), Shannon information index (*I*), observed heterozygosity (*H_o_*), Nei’s expected heterozygosity (*H_e_*), inbreeding coefficient (*F_is_*), fixation index (*F_st_*), inbreeding coefficient at the total sample level (*F_it_*), the polymorphism information content (PIC), Nei’s coefficient of genetic differentiation (*G_ST_*), Shannon’s coefficient of genetic differentiation (*G_ST_’*),gene flow (*N_m_*).

Loci	*N_a_*	*RA*	*I*	*H_o_*	*H_e_*	*F_is_*	*F_st_*	*F_it_*	PIC	*G_ST_*	*G_ST_’*	*N_m_*
LT4	10	9	0.6852	0.1752	0.2760	0.0771	0.3561	0.3740	0.2693	0.2290	0.2924	0.4520
LT7	3	0	0.8830	0.6674	0.5191	-0.5216	0.1303	-0.2798	0.4580	0.1081	0.2045	1.6684
LT9	7	5	0.8299	0.4062	0.4678	-0.1183	0.2440	0.1394	0.3984	0.2603	0.4019	0.7746
LT10	11	9	1.0898	0.9009	0.5817	-0.7451	0.1341	-0.5420	0.5040	0.0996	0.2128	1.6148
LT15	5	3	0.6333	0.2412	0.3537	0.0066	0.3141	0.3258	0.3087	0.2912	0.3919	0.5458
LT16	4	2	0.7875	0.9978	0.5203	-0.9328	0.0034	-0.9262	0.4052	0.0075	0.0157	72.932
LT25	11	5	1.8425	0.5351	0.8008	0.0490	0.2775	0.3129	0.7761	0.2736	0.6667	0.6507
LT28	4	1	0.7642	0.2975	0.4400	-0.0567	0.4180	0.3850	0.3824	0.3908	0.5387	0.3481
Mean	6.875	4.25	0.9394	0.5277	0.4949	-0.2802	0.2215	-0.0581	0.4378	0.2075	0.3406	0.8785

**Table 2:** Analysis of molecular variance (AMOVA) based on microsatellite loci in *L. tibetica.*

Source of Variation	Degrees of Freedom	Sum of Squares	Variance Components	Percentage of Variation	*p*-Value
**(i) total populations**					
Among populations	30	385.473	0.38671	18.3161	<0.001
Within populations	879	1497.408	1.72461	81.6839	<0.001
**(ii) two groups**					
Among groups	1	150.963	0.51530	20.9305	<0.001
Among populations within groups	29	234.510	0.22204	9.0187	<0.001
Within populations	879	1497.408	1.72461	70.0508	<0.001

## Supplementary Materials

Supplementary materials are available online at http://www.mdpi.com/2073-4425/10/2/97/s1. Table S1: Sample collection information, Table S2: Genetic characteristics of eight microsatellite loci in *L. tibetica* populations, Table S3: The pairwise values of *F_st_* and *N_m_* of *L. tibetica* populations, Figure S1: The results of Ln*Pr*(*X|K*) and Δ*K* after grouping in STRUCTURE.
