# Supplementary material for: Genetic Structure and Eco-Geographical Differentiation of Lancea tibetica in the Qinghai-Tibetan Plateau
Source: Genes (Basel). 2019 Jan 29;10(2):97. doi: 10.3390/genes10020097 (PMC6409646; doi:10.3390/genes10020097)

Table S1. Sample collection information

POP	Province	Location	Longitude	Latitude	Altitude（m）	No.	Voucher
LZ	Tibet	Luozha	28.15°	90.69°	4566	8	Chen2013394
XH	Qinghai	Xinghai	35.34°	99.91°	3622	12	Zhang2014089
AJL	Tibet	Anjiula	29.53°	96.78°	4140	6	Chen2014298
YD	Tibet	Yadong	27.79°	89.15°	4350	8	Chen2014498
TJ	Qinghai	Tianjun	37.20°	99.22°	3340	14	Zhang2014262
XHZ	Qinghai	Xihaizhen	36.87°	100.90°	3137	13	Zhang2014276
YS	Qinghai	Yushu	32.92°	97.23°	3667	18	Chen2014591
BT	Qinghai	Bangtang	32.78°	97.30°	4100	21	Chen2014602
ZD	Qinghai	Zhiduo	33.50°	96.10°	4370	19	Chen2014667
XLX	Qinghai	Xialaxiu	32.39°	96.80°	3770	16	Chen2014637
QML	Qinghai	Qumalai	33.97°	96.58°	4570	18	Chen2014684
MY	Qinghai	Menyuan	37.85°	101.08°	3636	30	Zhang2014341
DL	Qinghai	Dulan	37.03°	98.66°	3445	6	Zhang2014380
QL	Qinghai	Qilian	38.45°	99.57°	3296	22	Zhang2010431
GC	Qinghai	Gangcha	37.71°	100.57°	3442	15	Zhang2014300
MLS	Tibet	Milashan	29.71°	92.05°	4136	20	Zhang2014019
HK	Qinghai	Heka	35.87°	99.94°	3429	14	Zhang2014055
TR	Qinghai	Tongren	35.28°	101.92°	3036	8	Zhang2014151
GD	Qinghai	Guide	36.36°	101.44°	3782	14	Zhang2014188
GH	Qinghai	Gonghe	36.78°	99.68°	3396	12	Zhang2014378
DW	Qinghai	Dawu	34.48°	99.95°	3872	28	Zhang2015063
GAD	Qinghai	Gande	34.13°	100.31°	4020	16	Zhang2015094
DR	Qinghai	Dari	33.69°	99.44°	4028	7	Zhang2015107

HN	Qinghai	Henan	34.45°	101.05°	3657	10	Zhang2015145
HZ	Gangsu	Hezuo	34.84°	103.00°	3220	12	Fu2016012
BX	Tibet	Baxiu	30.20°	97.28°	4140	23	Fu2016184
SD	Sichuan	Seda	32.30°	100.28°	3926	12	Fu2016040
DX	Tibet	Dangxiong	30.54°	91.34°	4381	8	Fu2016210
LNZ	Tibet	Linzhou	30.08°	91.28°	4232	13	Fu2016208
JD	Qinghai	Jieduo	32.86°	95.00°	4327	19	Zhang2016753
ZQ	Qinghai	Zhaqing	33.08°	95.16°	4289	14	Zhang2016723

Table S2 Genetic Characteristics of eight microsatellite loci in *L. tibetica* populations. Observed number of alleles (N_a), effective number of alleles per locus (N_e), Shannon information index (I), the percentage of polymorphic loci (PPL), observed heterozygosity (H_o), Nei's expected heterozygosity (H_e).

POP	N_a	N_e	I	PPL	H_o	H_e
LZ	3.1250	2.4170	0.8751	87.50	0.6562	0.5059
XH	3.0000	2.0001	0.7341	100.00	0.4924	0.4301
AJL	2.1250	1.8436	0.5644	62.50	0.4792	0.3529
YD	2.750	2.2328	0.7898	87.50	0.5647	0.4700
TJ	2.500	1.7724	0.6137	100.00	0.5357	0.3782
XHZ	2.750	2.0601	0.6771	75.00	0.5769	0.3987
YS	2.8750	2.0479	0.7345	87.50	0.5748	0.4315
BT	2.5000	1.8283	0.6221	100.00	0.4975	0.3843
ZD	2.5000	1.9049	0.6401	75.00	0.5874	0.3921
XLX	2.3750	1.8871	0.6038	87.50	0.4932	0.3682
QML	2.3750	1.7709	0.5814	75.00	0.4783	0.3721
MY	2.8750	1.7612	0.6329	87.50	0.4609	0.3717
DL	1.7500	1.5903	0.4379	62.50	0.5208	0.2986
QL	2.8750	1.9382	0.7196	100.00	0.5381	0.4334
GC	2.2500	1.8297	0.5946	87.50	0.5054	0.3744
MLS	3.3750	2.2924	0.8637	100.00	0.6250	0.5091
HK	3.0000	1.8816	0.7335	100.00	0.5030	0.4232
TR	2.0000	1.7922	0.5945	87.50	0.6562	0.4043
GD	2.6250	1.6064	0.5600	100.00	0.4643	0.3393
GH	1.8750	1.4419	0.3763	75.00	0.4195	0.2453
DW	2.6250	1.5919	0.5213	100.00	0.4420	0.3220
GAD	2.6250	1.6499	0.6078	100.00	0.5234	0.3669
DR	2.3750	1.7355	0.6211	100.00	0.5536	0.3980
HN	2.1250	1.5357	0.4667	87.50	0.4375	0.2925
HZ	2.8750	1.9484	0.7530	100.00	0.5625	0.4505
BX	2.8750	1.7373	0.6258	87.50	0.4387	0.3595
SD	3.0000	2.0655	0.7695	100.00	0.6437	0.4632
DX	2.6250	2.0088	0.7216	87.50	0.5938	0.4326
LNZ	3.0000	2.1248	0.7526	87.50	0.6130	0.4407
JD	3.8750	2.0751	0.8298	100.00	0.5818	0.4607
ZQ	2.6250	1.9023	0.6419	87.50	0.5446	0.3871
Mean	2.6500	1.8798	0.6536	89.5	0.5343	0.3954

Table S3. The pairwise values of F_{st} (below diagonal) and N_m (above diagonal) of *L. tibetica* populations.

POP	LZ	AJL	YD	MLS	BX	DX	LNZ	XH	HK	GH	DL	TJ	QL	GC	XHZ	MY	YS	BT	ZD	XLX	QML	JD	ZQ	HZ	TR	GD	DW	GAD	DR	HN	SD
LZ		3.2465	2.9968	9.7500	0.8601	4.6138	2.5340	0.6641	0.6614	0.3035	0.3035	0.4842	0.6021	0.5872	0.5264	0.4393	0.5349	0.3995	0.4604	0.4700	0.4019	0.6079	0.4728	0.5814	0.5421	0.4264	0.3358	0.3691	0.4894	0.3414	0.5510
AJL	0.0715		2.2131	2.4181	0.8843	7.5625	2.4942	0.5853	0.5960	0.2722	0.2287	0.4501	0.6214	0.5567	0.4985	0.4441	0.5173	0.3700	0.4204	0.4921	0.3777	0.5743	0.4281	0.5406	0.4282	0.4128	0.3302	0.3256	0.4183	0.2819	0.4849
YD	0.0770	0.1015		2.4124	0.7708	4.6233	4.6713	0.7938	0.9248	0.4368	0.3687	0.6574	0.7831	0.6516	0.7346	0.5856	0.7564	0.5419	0.6832	0.7696	0.5678	0.8341	0.5767	0.6906	0.5836	0.6038	0.4305	0.4483	0.6477	0.4055	0.6211
MLS	0.0250	0.0937	0.0939		0.7544	4.8835	3.2271	0.7381	0.7921	0.4168	0.3789	0.5596	0.7358	0.7009	0.5992	0.5238	0.6121	0.4799	0.5620	0.5955	0.5014	0.6732	0.5497	0.6506	0.5867	0.5342	0.4177	0.4418	0.5850	0.4226	0.6085
BX	0.2252	0.2204	0.2449	0.2489		0.7805	0.7869	0.9630	1.1561	0.4899	0.3452	0.9577	1.0229	1.0288	0.9433	0.7239	0.8984	0.4791	0.5681	0.6722	0.5670	1.0596	0.6503	0.7327	0.9648	0.6588	0.4918	0.4620	0.6038	0.4154	0.6941
DX	0.0514	0.0320	0.0513	0.0487	0.2426		227.022	0.6678	0.6970	0.3402	0.2982	0.5103	0.7182	0.6208	0.5570	0.4703	0.5649	0.4183	0.4719	0.5689	0.4318	0.6384	0.4929	0.5943	0.5150	0.4831	0.3561	0.3679	0.5083	0.3303	0.5716
LNZ	0.0898	0.0911	0.0508	0.0719	0.2411	0.0011		0.7277	0.7613	0.4138	0.3303	0.5565	0.7149	0.6375	0.6050	0.5048	0.6076	0.4299	0.5281	0.6050	0.4918	0.6811	0.4954	0.6001	0.5526	0.5503	0.3999	0.4036	0.5518	0.3647	0.5707
XH	0.2735	0.2993	0.2395	0.2530	0.2061	0.2724	0.2557		5.8775	1.1297	0.7801	5.3304	3.0789	5.9228	7.0599	5.0691	2.6071	0.7658	3.6441	2.1040	2.8062	7.1684	1.1810	5.7166	2.6843	2.6368	2.4733	1.9821	2.4882	2.3955	1.5218
HK	0.2743	0.2955	0.2128	0.2399	0.1778	0.2640	0.2472	0.0408		3.1057	1.2866	16.7568	12.3763	3.7823	6.7923	8.5219	13.1190	1.3598	2.4039	5.9535	6.4885	6.7528	1.6584	3.6563	2.6604	7.8933	2.8402	1.9488	8.0833	1.6199	1.8669
GH	0.4517	0.4787	0.3640	0.3749	0.3379	0.4236	0.3766	0.1812	0.0745		0.9665	1.9239	1.9722	0.9074	1.6686	2.6706	3.0789	0.7891	1.0152	3.5207	3.6685	1.3047	0.6658	1.4034	0.7141	12.5051	2.4973	1.3434	4.2464	0.8596	0.8606
DL	0.4517	0.5222	0.4041	0.3975	0.4200	0.4560	0.4308	0.2427	0.1627	0.2055		1.1229	1.8740	0.6561	0.8276	1.0562	2.4011	1.4426	0.6641	1.1259	1.9258	1.2542	1.1358	1.4909	0.8084	1.1632	1.0589	1.2932	6.2267	0.6888	2.5402
TJ	0.3405	0.3571	0.2755	0.3088	0.2070	0.3288	0.3100	0.0448	0.0147	0.1150	0.1821		8.1112	2.9347	15.8790	5.5505	11.9451	1.2443	1.8264	4.3882	3.3784	8.9075	1.9565	3.4592	4.6328	4.0678	2.2010	1.5230	3.5094	1.4577	2.4733
QL	0.2934	0.2869	0.2420	0.2536	0.1964	0.2582	0.2591	0.0751	0.0198	0.1125	0.1177	0.0299		4.7103	3.7372	4.2545	8.1675	1.5708	1.5230	3.8150	3.2711	5.3933	2.7262	4.4142	3.7953	3.5962	2.1242	1.8281	8.6468	1.5722	4.5301
GC	0.2986	0.3099	0.2773	0.2629	0.1955	0.2871	0.2817	0.0405	0.0620	0.2160	0.2759	0.0785	0.0504		5.0466	5.1848	1.9391	0.6438	1.9802	1.3243	1.6925	2.3089	0.9155	5.4578	3.8417	1.8615	2.0996	1.9296	1.8922	1.8704	1.3067
XHZ	0.3220	0.3340	0.2539	0.2944	0.2095	0.3098	0.2924	0.0342	0.0355	0.1303	0.2320	0.0155	0.0627	0.0472		9.4025	5.0354	0.9560	3.9517	3.1102	3.3627	3.7693	1.0869	5.2688	4.2872	3.6932	2.7803	2.1401	2.5909	1.7452	1.4909
MY	0.3627	0.3602	0.2992	0.3231	0.2567	0.3471	0.3312	0.0470	0.0285	0.0856	0.1914	0.0431	0.0555	0.0460	0.0259		3.5844	0.7712	2.6302	2.5063	3.8283	2.4912	0.8542	11.9451	1.8922	11.7692	16.9914	5.6602	6.3117	5.7166	1.1648
YS	0.3185	0.3258	0.2484	0.2900	0.2177	0.3068	0.2915	0.0875	0.0187	0.0751	0.0943	0.0205	0.0297	0.1142	0.0473	0.0652		3.0569	1.8178	6.7138	6.6561	6.1603	2.5621	3.1012	3.0395	4.0828	2.0952	1.7139	10.9608	1.2661	3.8350
BT	0.3849	0.4032	0.3157	0.3425	0.3429	0.3741	0.3677	0.2461	0.1553	0.2406	0.1477	0.1673	0.1373	0.2797	0.2073	0.2448	0.0756		0.7729	1.6411	1.3184	1.5937	2.6570	0.8470	0.9695	0.8248	0.6121	0.6112	1.3861	0.4851	2.5247
ZD	0.3519	0.3729	0.2679	0.3079	0.3056	0.3463	0.3213	0.0642	0.0942	0.1976	0.2735	0.1204	0.1410	0.1121	0.0595	0.0868	0.1209	0.2444		1.9449	3.6502	2.1678	0.8271	2.1401	1.3125	1.5056	1.6411	1.7170	1.5551	1.2381	0.9047
XLX	0.3472	0.3369	0.2452	0.2957	0.2711	0.3053	0.2924	0.1062	0.0403	0.0663	0.1817	0.0539	0.0615	0.1588	0.0744	0.0907	0.0359	0.1322	0.1139		6.0157	3.2662	1.8076	1.6425	1.2688	3.9236	1.5564	1.0970	3.3575	0.9021	1.6171
QML	0.3835	0.3983	0.3057	0.3327	0.3060	0.3667	0.3370	0.0818	0.0371	0.0638	0.1149	0.0689	0.0710	0.1287	0.0692	0.0613	0.0362	0.1594	0.0641	0.0399		3.3890	1.3598	2.6774	1.5268	4.0235	2.9026	2.4039	13.0479	1.3744	1.6555
JD	0.2914	0.3033	0.2306	0.2708	0.1909	0.2814	0.2685	0.0337	0.0357	0.1608	0.1662	0.0273	0.0443	0.0977	0.0622	0.0912	0.0390	0.1356	0.1034	0.0711	0.0687		3.5785	2.8364	2.9883	2.0457	1.4380	1.3184	2.8947	1.2086	3.2222
ZQ	0.3459	0.3687	0.3024	0.3126	0.2777	0.3365	0.3354	0.1747	0.1310	0.2730	0.1804	0.1133	0.0840	0.2145	0.1870	0.2264	0.0889	0.0860	0.2321	0.1215	0.1553	0.0653		0.9913	1.6599	0.8134	0.6059	0.5926	1.3353	0.5044	8.0556
HZ	0.3007	0.3162	0.2658	0.2776	0.2544	0.2961	0.2941	0.0419	0.0640	0.1512	0.1436	0.0674	0.0536	0.0438	0.0453	0.0205	0.0746	0.2279	0.1046	0.1321	0.0854	0.0810	0.2014		2.5402	3.9236	8.0833	20.5833	9.6706	9.9541	2.0186
TR	0.3156	0.3686	0.2999	0.2988	0.2058	0.3268	0.3115	0.0852	0.0859	0.2593	0.2362	0.0512	0.0618	0.0611	0.0551	0.1167	0.0760	0.2050	0.1600	0.1646	0.1407	0.0772	0.1309	0.0896		1.1577	1.0494	1.1021	1.5642	0.8626	3.3890
GD	0.3696	0.3772	0.2928	0.3188	0.2751	0.3410	0.3124	0.0866	0.0307	0.0196	0.1769	0.0579	0.0650	0.1184	0.0634	0.0208	0.0577	0.2326	0.1424	0.0599	0.0585	0.1089	0.2351	0.0599	0.1776		11.6279	3.1928	16.0256	2.7563	1.4286
DW	0.4268	0.4309	0.3674	0.3744	0.3370	0.4125	0.3847	0.0918	0.0809	0.0910	0.1910	0.1020	0.1053	0.1064	0.0825	0.0145	0.1066	0.2900	0.1322	0.1384	0.0793	0.1481	0.2921	0.0300	0.1924	0.0215		13.4863	7.1905	13.5622	0.9193
GAD	0.4038	0.4343	0.3580	0.3614	0.3511	0.4046	0.3825	0.1120	0.1137	0.1569	0.1620	0.1410	0.1203	0.1147	0.1046	0.0423	0.1273	0.2903	0.1271	0.1856	0.0942	0.1594	0.2967	0.0120	0.1849	0.0783	0.0182		6.1933	8.8741	1.0114
DR	0.3381	0.3741	0.2785	0.2994	0.2928	0.3297	0.3118	0.0913	0.0300	0.0556	0.0386	0.0665	0.0281	0.1167	0.0880	0.0381	0.0223	0.1528	0.1385	0.0693	0.0188	0.0795	0.1577	0.0252	0.1378	0.0156	0.0336	0.0388		2.3515	2.9551
HN	0.4227	0.4700	0.3814	0.3717	0.3757	0.4308	0.4067	0.0945	0.1337	0.2253	0.2663	0.1464	0.1372	0.1179	0.1253	0.0419	0.1649	0.3401	0.1680	0.2170	0.1539	0.1714	0.3314	0.0245	0.2247	0.0907	0.0181	0.0274	0.0961		0.8080
SD	0.3121	0.3402	0.2870	0.2912	0.2648	0.3043	0.3046	0.1411	0.1181	0.2251	0.0896	0.0918	0.0523	0.1606	0.1436	0.1767	0.0612	0.0901	0.2165	0.1339	0.1312	0.0720	0.0301	0.1102	0.0687	0.1750	0.2138	0.1982	0.0780	0.2363	

Figure S1 The results of LnPr（$X|K$） and ΔK after grouping in STRUCTURE. (A)The results of Ln$Pr(X|K)$, (B)The results of ΔK

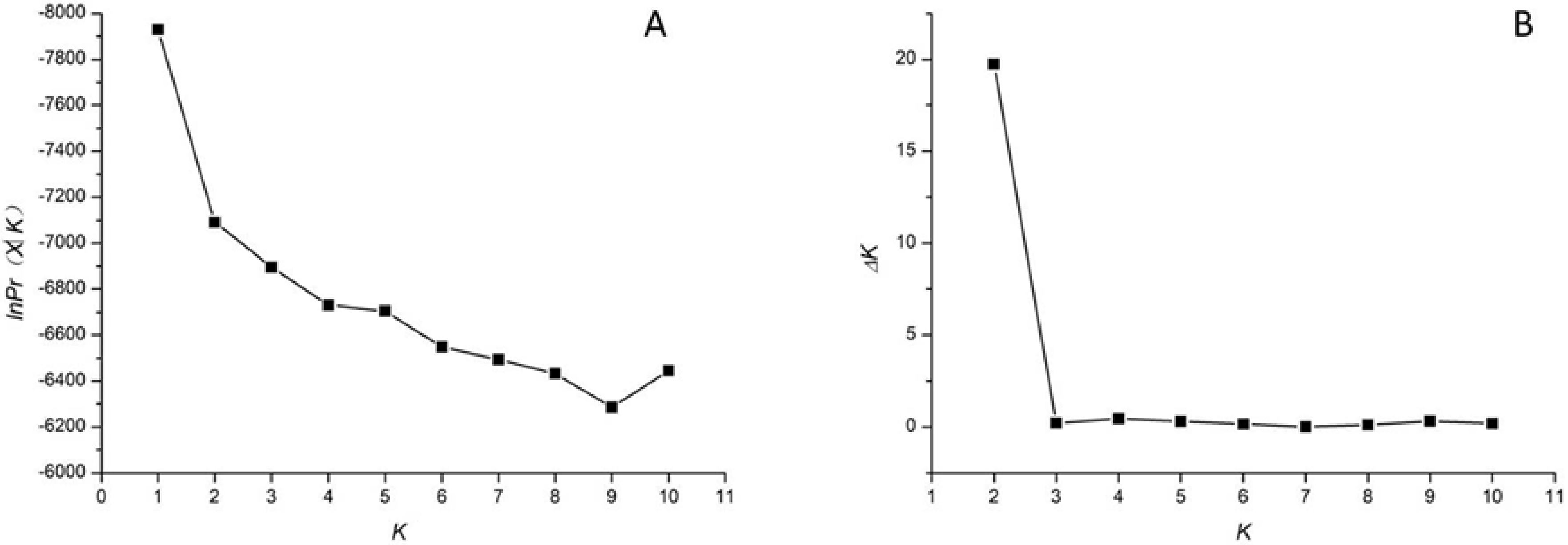

Supplement: Supplementary file 1 [file genes-10-00097-s001.pdf]
